# Clinical Factors Associated With Intracranial Aneurysms in Patients With Autosomal Dominant Polycystic Kidney Disease

**DOI:** 10.7759/cureus.97792

**Published:** 2025-11-25

**Authors:** Yutaka Fuchinoue, Yuki Sakaeyama, Ryo Matsuzaki, Shuhei Kubota, Mitsuyoshi Abe, Sayaka Terazono, Masaaki Nemoto, Nobuo Sugo

**Affiliations:** 1 Department of Neurosurgery, Faculty of Medicine, Toho University, Sakura, JPN; 2 Department of Neurosurgery, Faculty of Medicine, Toho University, Tokyo, JPN

**Keywords:** age, artificial dialysis, autosomal dominant polycystic kidney disease, intracranial aneurysm, screening

## Abstract

Introduction

Autosomal dominant polycystic kidney disease (ADPKD) is associated with an increased prevalence of intracranial aneurysms (IAs), but the clinical factors influencing IA development remain unclear. This study aimed to identify the clinical factors associated with the presence or absence of IAs in patients with ADPKD.

Methods

We conducted a retrospective cohort study of 138 Japanese patients with ADPKD who underwent neuroradiological imaging between January 2004 and December 2020. Patients were categorized into IA (n=30) and non-IA (n=108) groups. Clinical characteristics, including age at ADPKD diagnosis, dialysis status, comorbidities, and family history of IA, were compared using univariate and multivariate analyses. Correlation between age at IA diagnosis and age at dialysis initiation was also examined.

Results

The prevalence of IA was 21.7%, with multiple IAs in 30.0% of affected patients and subarachnoid hemorrhage (SAH) in 23.3%. The most common IA locations were the internal carotid artery (35.7%), middle cerebral artery (28.6%), and anterior cerebral artery (21.4%). Patients in the IA group were older at ADPKD diagnosis (54.5±13.5 vs. 46.8±13.7 years, p<0.01) and more frequently on dialysis (60.0% vs. 34.3%, p<0.05). Multivariate analysis identified older age at ADPKD diagnosis and dialysis use as independent factors associated with IA. Among dialysis patients, age at IA diagnosis correlated positively with age at dialysis initiation (r=0.885, p<0.0001).

Conclusion

IAs were detected in approximately one-fifth of ADPKD patients, particularly in those of advanced age and on dialysis. The statistically significant and positive correlation between IA diagnosis age and dialysis initiation suggests a relationship between IA development and renal function decline. Targeted IA screening, especially in elderly ADPKD patients and at the time of dialysis initiation, with periodic reimaging for high-risk individuals, may improve early detection and management.

## Introduction

Autosomal dominant polycystic kidney disease (ADPKD) is an inherited renal disease with a frequency of 1:1,000 and is associated with various extrarenal lesions and renal cysts [1,2]. Other organ lesions include liver and pancreatic cysts, aortic root dilatation and aneurysms, mitral valve prolapse, abdominal wall hernias, and intracranial aneurysms (IAs) [2,3]. The prognosis for subarachnoid hemorrhage (SAH) due to ruptured IA is particularly poor, with only 36% of patients in the independent activities of daily living state, 21% requiring assistance with daily living, and a 43% mortality rate [4]. Some studies reported that the rupture rate of IA in patients with ADPKD is higher than that in the general population, whereas other studies reported no difference in rupture rates [5,6]. Patients with ADPKD are weary about SAH due to ruptured IA and are afraid because it is devastating [7]. IA screening in patients with ADPKD is also controversial among specialists. While some reports recommend systematic screening for IA [8,9], others suggest screening only for patients with history of SAH, family history of IAs, high-risk occupations, or anxiety [2]. Therefore, more detailed investigation on the clinical characteristics of patients with IAs and the timing of IA occurrence in ADPKD could help select patients for screening [10,11]. Thus, this study aimed to determine the clinical factors of ADPKD with and without IAs.

## Materials and methods

This study was a retrospective cohort study. Eligibility criteria for participants were ADPKD patients aged 18 years or older who visited the Department of Nephrology at our institution between December 2020 and January 2004 and were neuroradiologically examined for the presence of IA. A total of 68 patients without imaging studies were excluded, out of a total of 138 patients. All were Asian (Japanese). The mean age of the patients was 48.5±14.0 years (range, 19-83 years) and the male-to-female ratio was 1:1. The IA group and non-IA group included 30 (21.7%) and 108 (78.3%) patients, respectively. At our institution, patients were diagnosed with IA using magnetic resonance imaging (MRA), three-dimensional computed tomography angiography (3DCTA), and digital subtraction angiography (DSA). The subjects included cases diagnosed with unruptured IAs at other institutions and those treated for SAH. Between the IA and non-IA groups, age at diagnosis of ADPKD, gender, hypertension, dyslipidemia, diabetes mellitus, smoking, alcohol consumption, dialysis, family history of IA, age at dialysis induction, and age at diagnosis of IA were compared. Family history was investigated retrospectively in second-degree relatives. Additionally, in the IA group the relationship between age at diagnosis of IA and age at dialysis induction was examined.

Chi-square or t-test, univariate and multivariate comparison, and regression analysis, was used to determine statistical difference, logistic regression, and correlations, respectively, between the two groups. This study was approved by the ethics committee of our institution (M23015, 21006). This study information was made publicly available on our institution’s website, and eligible patients were guaranteed the opportunity to opt-out.

## Results

In this study, the complication rate of IA was 30 cases (21.7%). Multiple IAs occurred in nine cases. Among the 30 cases, one patient (3.3%) had four IAs, one patient (3.3%) had three IAs, seven patients (23.3%) had two IAs, and 21 patients (70.0%) had a single IA. Multiple IAs were diagnosed in a single imaging study, with no de novo IAs. Of these, seven cases (23.3%) developed SAH. Of the 42 IAs, including multiple IAs, 15 (35.7%), 12 (28.6%), and nine (21.4%) occurred in the internal carotid (IC) artery, middle cerebral artery (MCA), and anterior cerebral artery (ACA), respectively, alongside four in the vertebral artery (VA), and 2 in the basilar artery (BA) (Table 1).

**Table 1:** Location of IA IA: Intracranial aneurysm, IC: internal carotid artery, ICPC: IC-posterior communicating artery, IC anch: IC artery-anterior choroidal artery, CS: cavernous sinus, ACA: anterior cerebral artery, A com: anterior communicating artery, A1: A1 segment, VA-PICA: vertebral artery-posterior inferior cerebellar artery, BA: basilar artery.

Major arterial locations of IA development	n (%)	Specific arterial sites of IA	n (%)
IC	15 (35.7%)	ICPC	11 (26.2%)
		IC anch	2 (4.8%)
		CS	1 (2.4%)
		Dissection	1 (2.4%)
MCA	12 (28.6%)	-	-
ACA	9 (21.4%)	A com	8 (19.0%)
		A1	1 (2.4%)
VA	4 (9.5%)	VA–PICA	3 (7.1%)
		Dissection	1 (2.4%)
BA	2 (4.8%)	-	-

At our institution, 30 IAs were measured using MRA, 3DCTA, and DSA with an average size of 5.0±2.0 (2.1-10.8) mm. A summary of cases for the IA and non-IA groups is shown in Table 2.

**Table 2:** Summary of cases IA: Intracranial aneurysm, IA group: group with intracranial aneurysm, non-IA: group without intracranial aneurysm, SD: standard deviation, M: male, F: female,  *p<0.05, **p<0.01.

	All	IA group	Non-IA group	Statistics	p-value	
n	138 (100%)	30 (21.7%)	108 (78.3%)			
Sex (n) (M:F)	69:69 (50%:50%)	14:16 (47%:53%)	55:53 (51%:49%)	χ²=0.17	0.68	
Age (mean±SD)	48.5±14.0	54.5±13.5	46.8±13.7	t=2.75	0.008	**
Hypertension (n) (yes, no)	83:55 (60%:40%)	18:12 (60%:40%)	65:43 (60%:40%)	χ²=0.00	1.000	
Dyslipidemia (n) (yes, No)	19:119 (14%:86%)	5:25 (17%:83%)	14:94 (13%:87%)	χ²=0.05	0.825	
Diabetes mellitus (n) (yes, no)	15:123 (11%:89%)	2:28 (7%:93%)	13:95 (12%:88%)	χ²=0.25	0.614	
Smoking (n) (yes, no)	42 :96 (30%:70%)	9:21 (30%:70%)	33:75 (31%:69%)	χ²=0.00	1.000	
Alcohol drinking (n) (yes, no)	11:127 (8%:92%)	2:28 (7%:93%)	9:99 (8%:92%)	χ²=0.00	1.000	
Family history of IA (n) (yes, no)	5:113 (4%:96%)	2:28 (7%:93%)	3:105 (3%:97%)	χ²=0.21	0.648	
Dialysis (n) (yes, no)	55:83 (40%:60%)	18:12 (60%:40%)	37:71 (34%:66%)	χ²=5.46	0.019	*
Hemodialysis (n)	50 (36%)	16 (53%)	34 (31%)	χ²=4.85	0.028	*
Peritoneal dialysis (n)	5 (4%)	2 (7%)	3 (3%)	χ²=1.02	0.313	
Renal transplantation	15(100%)	5(33%)	10(67%)	χ²=0.68	0.411	
Dialysis starting age (mean±SD)	54.6±10.3	55.0±12.5	52.5±10.6	t=1.00	0.323	

The average age at ADPKD diagnosis was higher in the IA group at 54.5±13.5 years than in the non-IA group (p <0.01), at 46.8±13.7 years. Additionally, 18 (60.0%) and 37 (34.3%) of patients in the IA group and non-IA group, respectively, were on dialysis (p<0.05). Univariate analysis with the presence of IA as the objective variable revealed significant differences between the age at ADPKD diagnosis and dialysis use (Table 3; p<0.05, p<0.01).

**Table 3:** Univariate analysis M: Male, F: female, CI: confidence interval, IA: intracranial aneurysm, *p<0.05, **p<0.01.

Explanation Variables	Partial regression coefficient	Odds ratio	95% CI (Lower limit)	95% CI (Upper limit)	p-value	
Sex (M0_ F1)	0.171	1.186	0.527	2.667	0.680	
Age	0.041	1.042	1.010	1.075	0.009	**
Hypetension (0_1)	-0.008	0.992	0.435	2.266	0.985	
Dyslipidemia (0_1)	0.295	1.343	0.442	4.084	0.603	
Diabetes mellitus (0_1)	-0.650	0.522	0.111	2.452	0.410	
Smoking (0_1)	-0.026	0.974	0.403	2.352	0.953	
Alcohol drinking (0_1)	-0.241	0.786	0.160	3.847	0.766	
Dialysis (0_1)	1.057	2.878	1.253	6.611	0.013	*
Family history of IA (0_1)	0.916	2.500	0.398	15.696	0.328	

Multivariate analysis also revealed statistically significant between the age at ADPKD diagnosis and dialysis use (Table 4; p<0.05).

**Table 4:** Multivariate analysis M: Male, F: female, CI: confidence interval, IA: intracranial aneurysm. *p<0.05.

Forcing in all variables							Forward-backward stepwise selection method					
Explanation Variables	Partial regression coefficient	Odds ratio	95% CI (Lower limit)	95% CI (Upper limit)	p-value		Partial regression coefficient	Odds ratio	95% CI (Lower limit)	95% CI (Upper limit)	p-value	
Sex (M0_ F1)	0.232	1.261	0.493	3.227	0.629		-	-	-	-	-	
Age	0.038	1.039	1.004	1.074	0.028	*	0.036	1.037	1.004	1.071	0.028	*
Hypetension (0_1)	-0.062	0.940	0.371	2.380	0.896		-	-	-	-	-	
Dyslipidemia (0_1)	0.573	1.774	0.489	6.436	0.383		-	-	-	-	-	
Diabetes mellitus (0_1)	-0.578	0.561	0.100	3.156	0.512		-	-	-	-	-	
Smoking (0_1)	-0.146	0.864	0.295	2.528	0.789		-	-	-	-	-	
Alcohol drinking (0_1)	0.184	1.202	0.195	7.425	0.843		-	-	-	-	-	
Dialysis (0_1)	0.910	2.484	1.030	5.991	0.043	*	0.862	2.368	1.008	5.561	0.048	*
Family history of IA (0_1)	1.260	3.525	0.470	26.4	0.220		-	-	-	-	-	
constant term	-3.777						-3.541					

**Figure 1:**
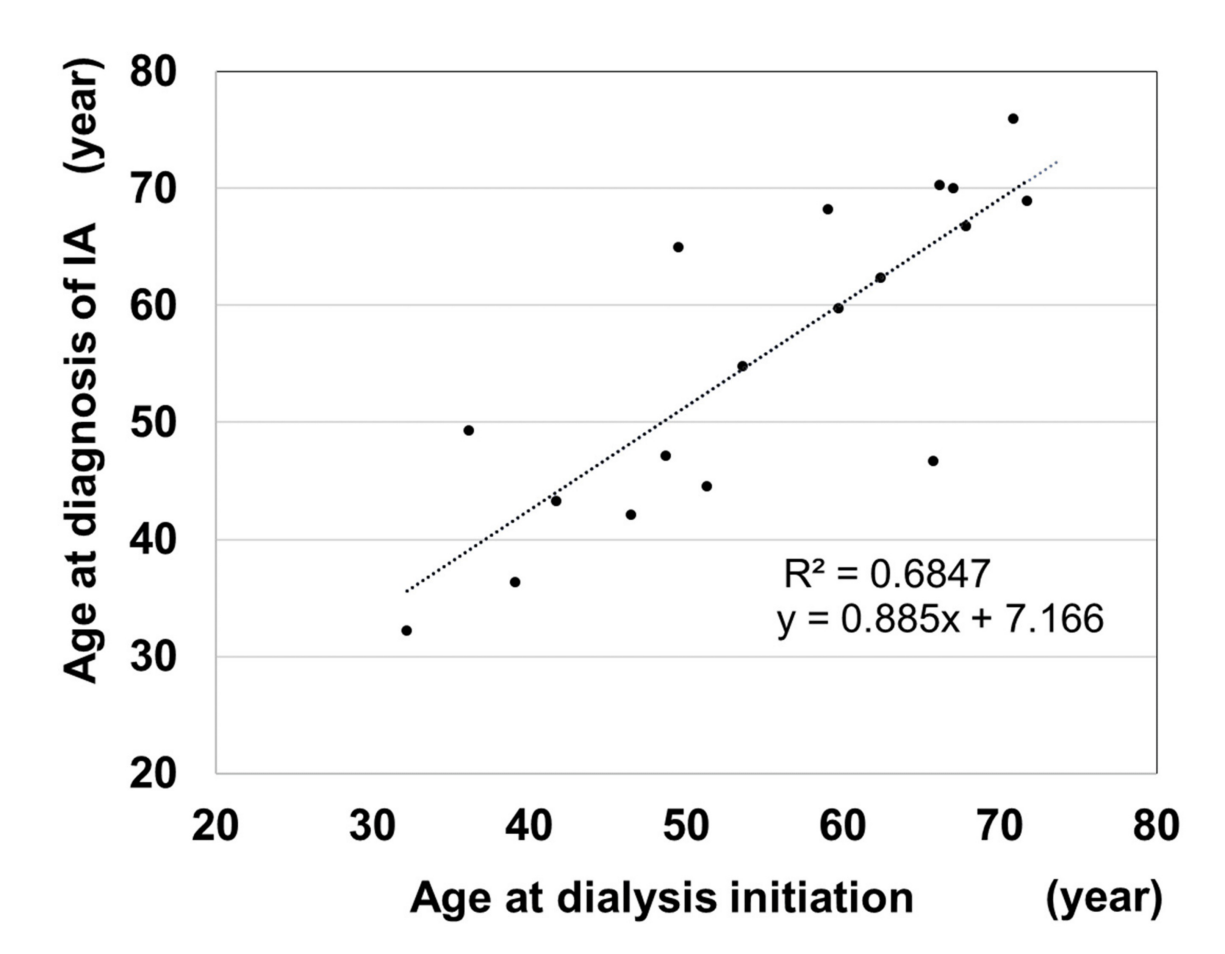
Correlation between age at diagnosis of intracranial aneurysm and age at dialysis initiation IA: Intracranial aneurysm.

When only dialysis patients were selected, only 18 patients in the IA group and 37 patients in the non-IA group were examined. The average age at dialysis initiation was similar between the IA group (55.0±12.5; 32-72 years) and the non-IA group (54.5±9.3; 36-75 years). Among patients on dialysis in the IA group, the average age at IA diagnosis was 55.8±13.3; 32-76 years, and the correlation between the age at dialysis initiation and age at IA diagnosis was statistically significantly and positive (Figure 1; y=0.885x + 7.166, R2=0.6847, p<0.0001).

## Discussion

In this study, the IA group had a higher age at diagnosis of ADPKD and more patients underwent dialysis than the non-IA group. In addition, age at IA diagnosis had a significant positive correlation with age at dialysis induction. According to postmortem autopsy data, the frequency of IA complications in ADPKD is 22.5% [5], which is four to five times higher than the general population [12]. Similarly, the prevalence of IA in ADPKD in this study was 21.7%. In the general population, the sites of IA occurrence are as follows: MCA, 36.2%; anterior communicating artery (A-com), 15.5%; IC, 18.6%; IC-posterior communicating artery (ICPC), 15.5%; BA, 6.6%; and VA, 1.8% [13]. IC, MCA, and A-com have also been reported as the most common sites for IA occurrence in ADPKD [4,14-16], and were also the most common sites in this study. IA locations associated with ADPKD may not have a characteristic distribution. The frequency of multiple IAs in ADPKD has been reported as 52.2% [14], 45% [10], and 30.3% [17], which is 25.1%-27.5% higher than the general population [13] and similar to this study’s population at 30%. The IA size in ADPKD has been reported as 3.85±3.25 mm [16], 4.5±2.7 mm [14], <9 mm [15], and 5.0±2.0 mm in this study, which is relatively small. All the patients in this study were Japanese, and there did not appear to be any particular clinical differences in the location, size, or multiplicity of IAs associated with ADPKD.

One of the risk factors for IA complications in ADPKD is a family history of IA [18]. The prevalence of IA in patients with a family history of IA or hemorrhagic stroke was reported to be as high as 21.6% and as low as 11.0% in patients who were negative for these conditions [16]. Moreover, the estimated lifetime risk of SAH due to IA in ADPKD is reported to be >20% when two or more first-degree relatives are affected by SAH due to IA [19].

Age is another risk factor for IA complications in ADPKD, and several characteristics have been reported. In studies of the general population, the average age of patients with SAH due to IA rupture is 57 years [20], whereas those in ADPKD with SAH are younger at 41 years [7]. Other studies reported that SAH due to IA rupture in ADPKD often occurred at a young age [4,10,21-23], and 64% of patients were younger than 50 years [5]. Conversely, a study including patients with ADPKD revealed that the prevalence of IA increased with increasing age, peaking at 23.3% in those aged 60-69 years [16]. Similarly, the prevalence of IA in patients with ADPKD was reported to be 2.9% in those younger than 45 years of age, compared with 22.4% in those older than 45 years of age [15]. In the present study, the age at ADPKD diagnosis was also higher in the IA group than in the non-IA group, indicating that advanced age is a risk factor for the occurrence of IA. One possible reason for the high incidence of IA in elderly patients with ADPKD is de novo IA, which is newly developed during the follow-up period. Belz et al. reported that five of 20 patients with ADPKD with a history of IA developed new IA during a follow-up period of 6-33 years [21]. Nakajima et al. found IA in three of 15 patients with ADPKD, and repeat MRA during 18-72 months showed the appearance of new IA in two patients [24]. Schrier et al. followed 76 patients with ADPKD for an average of 9.8 years and found that IA occurred in two patients with a family history of IA [25]. These reports demonstrate that IA in ADPKD is more likely to rupture at younger ages than in the general population, IA increases with age in the ADPKD population, and de novo IA is more likely to occur.

Whether renal dysfunction is a risk factor for the development of IA associated with ADPKD remains unclear [15,16,26]. A study examining chronic kidney disease between stages 1 and 5 and the prevalence of IA found a statistically significant difference only between stages 2 and 4 [15], while another has reported that the frequency of IA is unrelated to renal dysfunction [16]. In contrast, a retrospective study of 77 patients with IA in 64 families with ADPKD found that end-stage renal disease (ESRD) was common in 29 (37.7%) patients [26]. In the present results, the IA group was more likely to receive dialysis than the non-IA group, indicating the involvement of renal dysfunction as an IA complication. A complicating factor in the relationship between IA complications and renal dysfunction may vary in the timing of ESRD in individual cases of ADPKD. In ADPKD, the time of ESRD requiring dialysis or transplantation is typically in the 60s; however, some patients with ESRD still had adequate renal function well into their 90s, while some had enlarged kidneys in the neonatal period, which is also characterized by variability in the timing of ESRD. This is because ADPKD is genotypically heterogeneous [27]. Two causative genes, PKD1 and PKD2, have been identified in ADPKD at an occurrence of 75.5% and 18.3%, respectively [28]. Comparing the kidney prognoses of the two groups, a difference of approximately 20 years was observed, with PKD1 averaging 58.1 years and PKD2 averaging 79.7 years [28]. On correlating age at dialysis initiation with ESRD, we found a significantly positive correlation between age at IA diagnosis and age at dialysis initiation. In summary, the correlation between these two variables would indicate that IA complications are related to the timing of renal function deterioration. Therefore, based on the results, the time of dialysis induction may be recommended to screen for IA in patients with ADPKD. Based on these findings, IA screening is recommended for patients with ADPKD who are elderly, on dialysis, or at the dialysis induction stage. Periodic reimaging is recommended for these patients.

There are several limitations to this study. The fact that all subjects in this study were Japanese may limit the generalizability of the results. With its retrospective design and relatively small number of cases, this study may have been limited in statistical power. The age at diagnosis of IA in this study did not necessarily coincide with the time of occurrence of IA, which may be a limitation of a retrospective study. In addition, the finding that patients with IA had a higher age at diagnosis of ADPKD may reflect a delay in the detection of IA or the severity of the disease. Future validation in a prospective study with a population of ADPKD patients who regularly undergo head MRA or 3DCTA is desirable. In addition, it is recommended to examine the relationship between renal dysfunction and IA in blood tests as well as the presence or absence of dialysis. Herein, only patients with ADPKD who underwent neuroradiological imaging were included. Therefore, a bias of not including all patients with ADPKD who visited our institution exists.

## Conclusions

In this study, the development of IAs in patients with ADPKD was suggested to be influenced by renal function decline, the timing of progression to end-stage renal disease, and aging. These findings indicate that targeted screening and periodic reimaging in high-risk individuals - such as elderly patients, those receiving dialysis, or at the initiation of dialysis - may be useful for early detection and appropriate management. Furthermore, future research should address genetic investigations and the need for long-term follow-up to better clarify the mechanisms underlying IA development and optimize patient care.
